# Tablet Use Affects Preschoolers’ Executive Function: fNIRS Evidence from the Dimensional Change Card Sort Task

**DOI:** 10.3390/brainsci11050567

**Published:** 2021-04-29

**Authors:** Hui Li, Dandan Wu, Jinfeng Yang, Jiutong Luo, Sha Xie, Chunqi Chang

**Affiliations:** 1 School of Education, Macquarie University, Sydney, NSW 2109, Australia; dandan.wu4@students.mq.edu.au; 2 School of Biomedical Engineering, Health Science Center, Shenzhen University, Shenzhen 518061, China; yangjinfeng2017@email.szu.edu.cn; 3 Center for Educational Science and Technology, Beijing Normal University at Zhuhai, Zhuhai 519085, China; jtluo@bnu.edu.cn; 4 Normal College, The GBA Institute of Educational Research, and The Institute of KEEP Collaborative Innovation, Shenzhen University, Shenzhen 518061, China; xiesha@szu.edu.cn

**Keywords:** pad use, executive function, fNIRS evidence, Dimensional Change Card Sort (DCCS) task, preschoolers

## Abstract

This study aims to examine the impact of heavy use of tablets on preschoolers’ executive function during the Dimensional Change Card Sort (DCCS) task using the functional near-infrared spectroscopy (fNIRS). Altogether, 38 Chinese preschoolers (*M_age_* = 5.0 years, *SD* = 0.69 years, 17 girls) completed the tasks before the COVID-19 lockdown. Eight children never used tablets, while 16 children were diagnosed as the ‘heavy-user’. The results indicated that: (1) the ‘non-user’ outperformed the ‘heavy-user’ with a significantly higher correct rate in the DCCS task; (2) the two groups differed significantly in the activation of the prefrontal cortex (BA 9): the ‘non-user’ pattern is normal and healthy, whereas the ‘heavy-user’ pattern is not normal and needs further exploration.

## 1. Introduction

Rapidly advancing information and communication technologies (*ICT*) have nurtured a brand-new generation of ‘digital children’ in this ‘digital age’. Infants and young children are exposed to more technologies than before as they have more devices and apps readily available for their use, resulting in increased screen time and tablet use [1]. This phenomenon has worried public health organizations, parents, and scholars who are seriously concerned about the benefits and damages of tablet use in the early years (ages 0–5) [2]. So far, many studies have examined the impact of tablet use on young children’s brain development, but the results are still mixed [3]. These inconclusive results have created difficulties in making policies for early ICT use and education and have caused heated debates between the advocates and dissenters of the ‘digital child’ and tablet use [4]. Therefore, there is an urgent need for rigorous neuroimaging evidence to settle the debate. To fill this research gap, this article explored the impact of heavy use of tablets on young children’s executive function in the prefrontal cortex using functional near-infrared spectroscopy (fNIRS).

### 1.1. Tablet Use and Early Childhood Development

Touchscreen tablet computers, hereafter referred to as ‘tablets’, are lightweight, mobile devices with a flat, panel screen used for both display and input [5,6]. Tablets feature multimedia playback, digital photography, multitouch interface, long battery life, instant start-up, and wireless Internet connectivity, thus have been easily and frequently used by young children as tools for entertainment and education [7]. A recent study found that tablets were especially popular for viewing videos, learning, and gaming in young children, and the number of users increased with age [8]. For instance, according to Lawrence [9], about 80% of top-selling paid educational apps in Apple’s App Store were marketed for young children’s consumption in 2017. Recently, the COVID-19 lockdown in 2020 has caused a sudden increase in tablet use among preschoolers, who had to do online learning at home [7].

Tablet use in the early years might have posited both benefits and risks in early childhood development, thus deserves empirical studies. Rocha and Nunes reviewed 11 studies and found that the damages were superior to the benefits, especially when screen time increased [2]. They also underscored the difficulties in finding studies directed to the desired age and type of electronic device, which is a potential cause of bias. Small et al. conducted another systematic review and reported harmful effects such as heightened attention-deficit symptoms, impaired emotional and social intelligence, technology addiction, social isolation, impaired brain development, and disrupted sleep [3]. Meanwhile, they also found that various apps, videogames, and other online tools might benefit brain health in adulthood. However, no neuroimaging studies have explored tablet use’s impact on young children’s executive function, as indicated by the following review.

### 1.2. Neuroimaging Studies on Early Executive Function

Executive function (EF) refers to the brain’s specific cognitive, transactional, self-regulating functions that can control and direct one’s attention amid distractions, regulate emotional reactivity, inhibit behavior from responding to environmental demands and fulfill goals [10,11]. Miyake et al. identified three core components of EF: inhibition, working memory, and cognitive shifting [12]. The Dimensional Card Change Sort (DCCS) task requires inhibition, working memory, and cognitive shifting to complete the card sorting, thus providing insights into the wide spectrum of EF processes and the associated neural changes in early childhood [13].

However, young children might have difficulties in taking functional magnetic resonance imaging (fMRI) because they cannot stay still in the tube for a long time. Therefore, very few neuroimaging studies had explored preschoolers’ EF during the DCCS task until Moriguchi and Hiraki conducted the fNIRS study for the first time. This success has inspired more fNIRS studies to examine EF using the DCCS task [14]. The results jointly indicated that the prefrontal area was substantially involved in the cognitive shifting [15]. Recently, Xie et al. found that the children in the DCCS pass group (with cognitive shifting) significantly activated the prefrontal cortex than those in the perseverate group who did not complete the cognitive shifting, and the activation in the prefrontal region was significantly correlated with children’s executive function [10]. Very recently, Li et al. proposed and confirmed the ‘V-shape curve’ theory by identifying a significant decrease–increase cycle in BA 9, the neural correlate of cognitive shifting [11]. In conclusion, the existing fNIRS studies have jointly confirmed the prefrontal cortex (e.g., BA 9), which is also the region of interest in this study, was the neural correlate of cognitive shifting during the DCCS tasks.

EF develops rapidly during the preschool years and is sensitive to environmental influences and learning experiences [16]. Since the early 2010s, tablet use has become increasingly prevalent in young children’s daily life and has prompted substantial public concerns about its impact on children’s brain development [17,18]. Most of the existing studies have reported survey and behavioral evidence [2,3], yet no neuroimaging evidence has been reported. This study was thus dedicated to filling this research gap by conducting the DCCS task with fNIRS to understand the impact of heavy use of tablets on EF. Accordingly, the following research questions guided this study.

1. Will the heavy users perform significantly differently from the non-users in the DCCS task?
2. What are the significant differences in prefrontal activation as evidenced by fNIRS between the heavy-users and non-users in the DCCS task?

## 2. Materials and Methods

### 2.1. Participants

Originally, 64 right-handed Chinese preschoolers and their parents consented to participate in this study. All the parents of participating children provided written consent and were informed verbally of the study’s purpose and the fNIRS experiments’ safety. The University Ethics Committee approved the experiments. Because of the unexpected COVID-19 lockdown, only 42 of them completed this study. Four failed to complete the experiments, thus were excluded from this study, resulting in a final sample of 38 children (ages 4 to 6.3 years, *M_age_* = 5.0 years, *SD* = 0.69 years, 17 girls, 21 boys). A post hoc power analysis was conducted on G*Power 3.1 (https://www.psychologie.hhu.de) to test the difference between two independent group means, using a two-tailed test, a medium effect size (d = 0.50), and an alpha of 0.05. The result showed that the *t*-test between heavy-users and non-users groups could achieve a power of 0.32. This limited statistical power was caused by the modest sample size in the present study (N*_total_* = 38), thus might have limited the significance of some of the statistical comparisons conducted.

### 2.2. Measures

#### 2.2.1. Home Learning Environment and Practice Survey (HLEP)

This survey was used to collect information about young children’s formal and informal learning activities at home. HLEP was developed from the Home Literacy Environment Index (HLEI) [19] and modified for early learning in the Chinese contexts [20]. HLEP had 29 questions about family background, household income, parental education level, occupation, home language, and learning environment, home learning activities, and bilingual parenting (TV watching time, parent–child talk time, storytelling time, parent–child shared reading time, the teaching of Chinese or English reading and writing, early bilingual literacies and so on). Some items were rated on a 5-point scale, while most asked for the real number of books, hours, frequencies, or ages, such as frequency of reading to the child per week, quantity, and variety of books in Chinese. In particular, four questions were asked about the child’s tablet use at home. “Q15: Is your child allowed to use tablets (smartphone) at home? (yes; no)”, “Q16: What does your child do with tablets? (1) watch cartoons or videos; (2) listen to music; (3) play game; (4) chat with friends or others; (5) others”, “Q17: Your child’s total screen time is ___ minute/day”, and “Q18: When does your child use tablets? (1) bedtime; (2) time assigned by the parent; (3) time chosen by the child; (4) when parents are busy”. If the answer to Q15 is “no” and that to Q17 is “0 or Not/Applicable”, the child will be regarded as ‘non-user’ of tablets. Accordingly, eight children were included in the ‘non-user’ group (2 girls, 6 boys). Among the 30 tablet users, 16 (12 girls, 4 boys) were classified into the ‘heavy-user’ group because: (1) their daily screen time was more than the mean level (M = 17.98 min, SD = 14.29); (2) their use was neither controlled nor limited; and (3) they did multiple activities with tablets. In addition, the rest was classified into the ‘low-user’ group (*n* = 14, 3 girls, 11 boys), thus was removed from the comparative analysis.

#### 2.2.2. The Dimensional Change Card Sort (DCCS) Tasks

**Figure 1:**
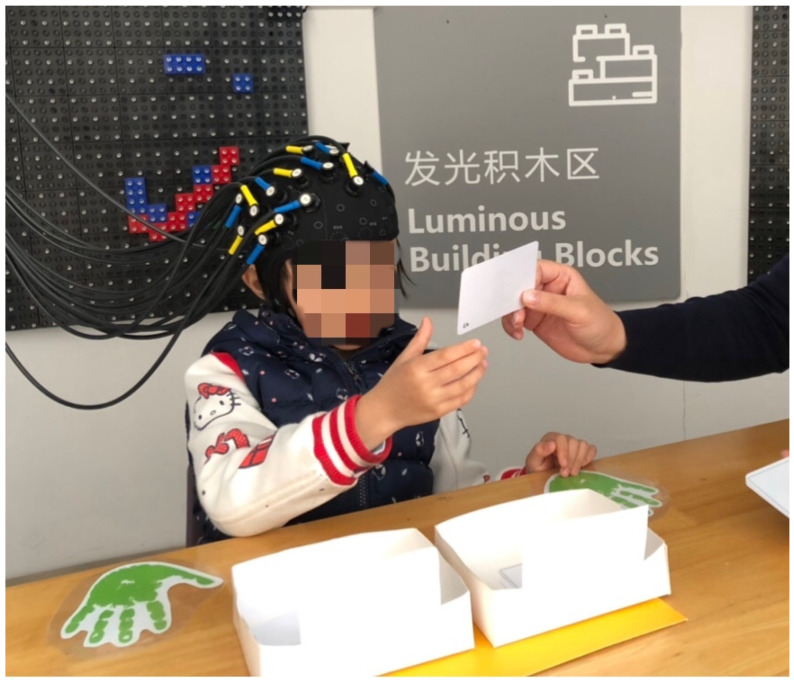
The Dimensional Change Card Sort (DCCS) tasks.

**Figure 2:**
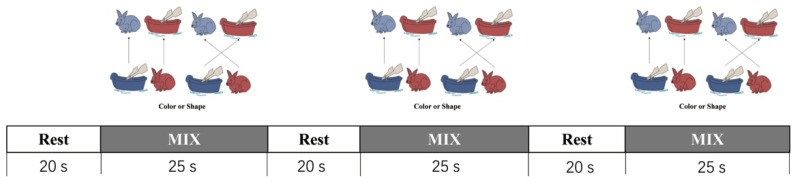
The DCCS task paradigm.

A set of white paper cards (3.5 cm × 7.0 cm) were used as the stimuli. The stimuli had two dimensions: shape and color. The DCCS task included target cards and test cards, which were matched in one dimension but did not match the other dimension (e.g., a red boat, a blue rabbit). Further, the rule for matching was changed according to the experimenter’s instruction. The present experiments included two target cards and 18 test cards, each of which was different in shape and color. One pair of target trays was used for the three consecutive test sessions, as shown in Figure 1. Each session consisted of a rest (20 s) phase and mix (25 s) phase, as shown in Figure 2. During the rest phase, children were asked to be still, doing nothing. During the mix phase, the children were asked to sort the cards according to the instructed rule (color or shape). In each phase, the children were given the rule before each trial. The rule order was fixed: shape, shape, color, shape, shape, color, shape, shape. The percentage of correct responses for each subject was recorded and analyzed. Each participant underwent a training session and three rounds of DCCS tasks. The training included six trials and allowed correction when children misunderstood the rules.

#### 2.2.3. The fNIRS Examination

**Figure 3:**
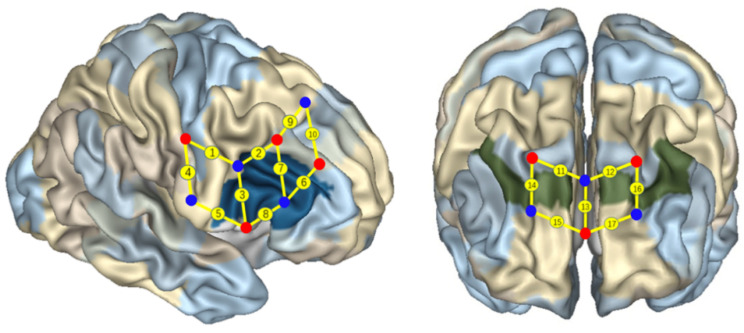
Localization of regions of interest.

In this study, a multiple-channel fNIRS system (Oxymon Mk III, Artinis, The Netherlands) was used to simultaneously measure the concentration changes of oxygenated hemoglobin (HbO), deoxygenated hemoglobin (HbR), and total hemoglobin (HbT) in the participants. Two wavelengths in the near-infrared range (i.e., 760 and 850 nm) were used to measure the changes in optical density and then converted into changes in the concentration of HbO and HbR using the modified Beer–Lambert law. The 17 channels were located following the international 10/20 system for EEG, with a 2.5 cm distance between each paired emitters and detectors. The region of interest (ROI) was located at Brodmann areas (BAs) 6/8/9/10/40/44 (Figure 3). Previous studies have shown that these areas were involved in EF in preschool children [10,11]. In particular, channels 1 and 9 were located in BA 6, channels 13, 15, 17 were located in BA 10, channel 10 was located in BA 8, channels 11, 12, 14, 16 were located in BA 9, channel 4 was located in BA 40, and channels 2, 3, 5, 6, 7, and 8 were located in the right IFC (BA 44).

### 2.3. Procedure

The study was conducted according to the guidelines of the Declaration of Helsinki, and was approved by the Ethics Committee of Shenzhen University (No. 2018005; January 2018).

#### 2.3.1. Data Collecting

The child caps accompanied with the NIRS instrument have digitized the optode positions. Both S and XS size of NIRS caps were used in this study to fit the head size of Chinese preschoolers. An experienced NIRS technician conducted cap placement, hair manipulation and tossing, and optodes installation (based on the 10/20 system). This process usually took 10 min, during which the participant was engaged in story-book reading with an experienced preschool teacher.

#### 2.3.2. Data Processing

A subject-specific differential path-length factor (DPF) constant was calculated based on each subject’s age, and the sampling rate was set at 50 Hz for data acquisition [21]. Each child’s DPF value was calculated according to the formula (DPF = 4.99 + 0.0678 Age^0.814^), which is more conducive to the data’s accuracy. The trials that contained deformity or noisy data were treated as incorrect trials and were discarded in advance of the formal analysis. The raw optical intensity data series were converted into changes in optical density (OD). The discrete wavelet transform was applied to every channel data series to remove motion artifacts, with the tuning parameter (α) of wavelet filtering set at 0.1. The bandpass filter (third-order Butterworth filter) with cut-off frequencies of 0.01–0.3 Hz was applied to reduce slow drifts and high-frequency noise. The OD data were then converted into concentration changes of HbO and HbR.

#### 2.3.3. Data Analysis

HbO and HbR concentration was converted into z-scores and calculated in the following analysis. Individual data were processed using MATLAB 2013b (Mathworks, MA, USA) and analyzed using the Homer2 NIRS processing package. The mean of z-scores (HbO and HbR) was calculated for each DCCS task block separately for each participant. Then, the mean of z-scores (HbO and HbR) was calculated by averaging across the three task blocks for each participant. Finally, the mean of z-scores (HbO and HbR) across all channels were compared using *t*-tests between ‘non-user’ and ‘heavy-user’ groups using SPSS. The General Linear Model (GLM) analysis predicting z-scores (HbO and HbR) in channel 16 was conducted in *R* (*Y_∆HbO_ = aX_time_ + b + ε*).

## 3. Results

### 3.1. Behavioral Results

We compared the ‘non-user’ and ‘heavy-user’ groups’ behavioral performance in the DCCS task (See Table 1). First, *t*-tests indicated that there were no significant differences in age between the ‘non-user’ (*M_Non-user_* = 5.03, *SD* = 0.41) and ‘heavy-user’ (*M_Heavy-user_* = 4.80, *SD* = 0.68) groups, *t* = 0.85, *p* > 0.001. Second, all the ‘non-user’ children passed all the testing items, *M_correct rate_* = 1. In contrast, not all the 16 ‘heavy-user’ children passed all the testing items, *M_correct rate_* = 0.922, S*D* = 0.14. The *t*-test results indicated that the ‘non-user’ group significantly outperformed the ‘heavy-user’ group, *t* = 2.256, *p* < 0.05. 

### 3.2. fNIRS Results

**Figure 4:**
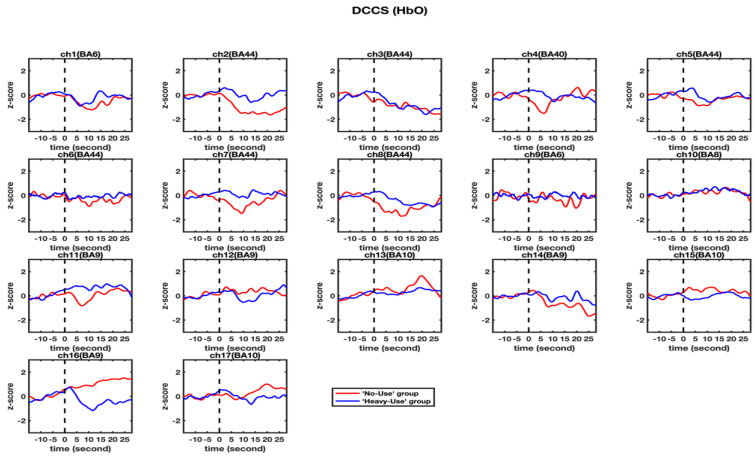
Observed changes in the HbO concentration in the 17 channels during the DCCS tasks. The HbO data for the ‘non-user’ and ‘heavy-user’ group are shown in red and blue line, respectively.

First, a set of independent-samples *t*-tests were conducted to determine whether there were significant differences in HbO increases in the 17 channels between the ‘non-user’ and ‘heavy-user’ groups. As multiple channels were involved in this type of *t*-tests, all the results were corrected for multiple comparisons using the false discovery rate (FDR), and the adjusted significance level of *p*-value was set at 0.05. As shown in Table 2 and Figure 4, significant differences were found only in BA 9 (ch 16) (*t* = 2.285, *p* < 0.05) between the ‘non-user’ (*M_HbO_* = 1.17, *SD* = 2.02) and the ‘heavy-user’ (*M_HbO_* = −0.62, *SD* = 1.70) groups. This result indicated that the ‘non-user’ group had significantly more activation in BA 9 than the ‘heavy-user’ group.

**Figure 5:**
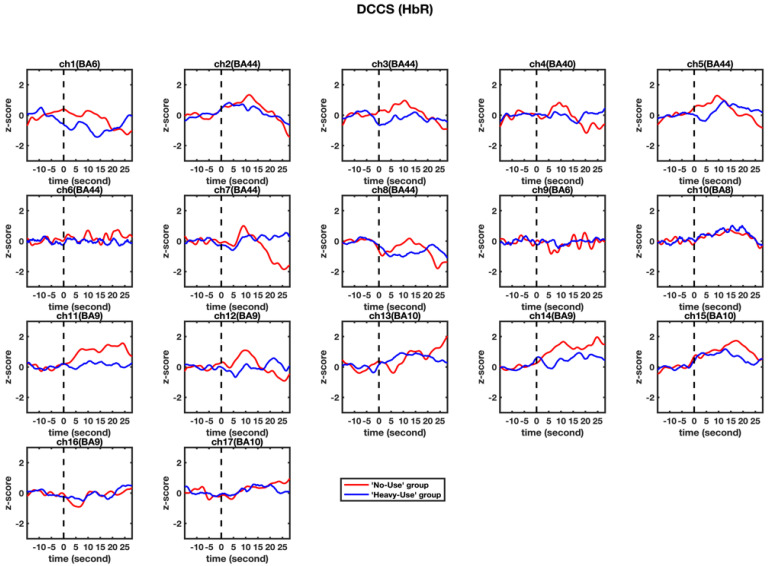
Observed changes in the HbR concentration in the 17 channels during the DCCS tasks. The HbR data for the ‘non-user’ and ‘heavy-user’ group are shown in red and blue line, respectively.

Second, a set of independent-sample *t*-tests were conducted to determine whether there were significant differences in HbR increases in the 17 channels between the ‘non-user’ and ‘heavy-user’ groups. As shown in Table 3 and Figure 5, no significant differences were found in any channel, *t*s < 1.441, *p*s > 0.164, after corrected with FDR.

**Figure 6:**
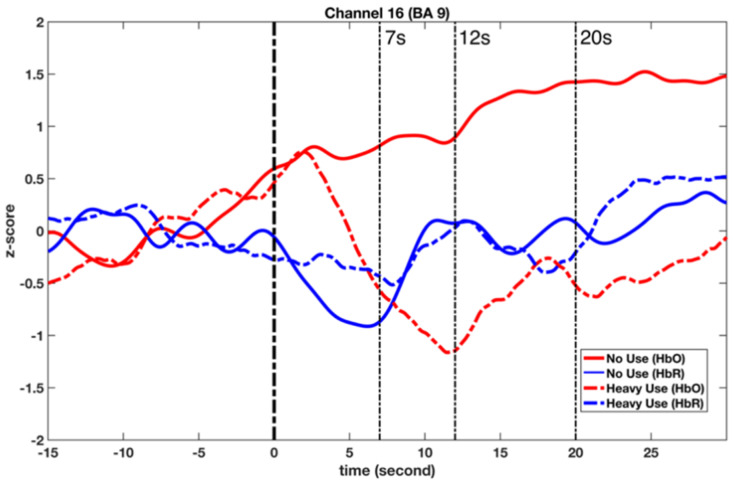
Observed changes in the HbO and HbR concentration in BA 9 (ch 16) during the DCCS tasks.

Third, a set of GLM analyses were conducted to model the changes in HbO in channel 16 based on experiment time for the ‘non-user’ and ‘heavy-user’ groups, respectively. As shown in Table 4 and Figure 6, during the DCCS task, significant HbO increase was observed in BA 9 (channel 16) for the ‘non-user’ group, *β* = 0.94, Δ*R*^2^ = 0.89, *F* = 803.45 (for the model), *t* = 28.35 (for *β*), *p* < 0.001. In contrast, significant decreases were found in BA 9 (channel 16) for the ‘high-use’ group, *β* = −0.33, Δ*R*^2^ = 0.10, *F* = 12.17 (for the model), *t* = −3.49 (for *β*), *p* < 0.01. However, after the 12th second, there was an increase in HbO in the ‘heavy-user’ group. These results indicated that BA 9 was significantly activated in the ‘non-user’ group during the DCCS task.

Last, a set of GLM analyses was conducted to model the changes in HbR in Channel 16 based on experiment time for the ‘non-user’ and ‘heavy-user’ groups, respectively. As shown in Table 5 and Figure 6, during the DCCS task, significant HbR increase was observed in BA 9 (channel 16) for both ‘non-user’ [*β* = 0.76, Δ*R*^2^ = 0.58, *F* = 134.99 (for the model), *t* = −3.49 (for *β*), *p* < 0.01] and ‘heavy-user’ groups [*β* = 0.81, Δ*R*^2^ = 0.65, *F* = 182.73 (for the model), *t* = 13.52 (for *β*), *p* < 0.01]. In particular, a significant decrease in HbR could be observed in the ‘non-user’ group before the 7th second. A significant decrease was observed in the ‘heavy-user’ group during 12th to 20th second.

## 4. Discussion

### 4.1. ‘Non-User’ Outperformed ‘Heavy-User’ Group in the DCCS

This study found that the young children who never used tablets had a 100% correct rate in the DCCS task, significantly outperforming those ‘heavy-users’. This finding is consistent with Horowitz–Kraus and Hutton [22], who found that increased screen time was associated with poorer executive functioning in very young children. In a systematic review, Small et al. also found that frequent tablet use might negatively affect brain function and behavior [3]. All these findings have jointly confirmed that tablet use has adversely impacted young children’s executive function. For the first time, this study provides fNIRS evidence to demonstrate the negative impact of tablet use, as discussed in the next section.

### 4.2. Two Activation Patterns in BA 9

This study found two distinctive activation patterns in BA 9 when processing the DCCS tasks. First, the ‘non-user’ pattern features a significant increase in HbO and a significant decrease in HbR before the 7th second during the task. This indicates a typical activation of BA 9, as an increase in HbO and a decrease in HbR are required to make a functional activation. Although the link to blood flow is only indirect and should be treated with care, this paired increase–decrease change in HbO and HbR demonstrates a complete hemodynamic picture. In fNIRS research, HbO is mainly linked to the tissue’s oxygen inflow, while HbR is linked to the amount of oxygen absorbed by the tissue [23]. In homeostasis, both the inflow of HbO and the formation of HbR should be constant, as the amount of oxygen being consumed by the tissue is equal to the amount of oxygen being carried towards the tissue. During the activation of BA 9, oxygen is consumed within the tissue, and hemodynamically, the tissue responds by increasing blood flow toward that area [24]. Therefore, this ‘non-user’ activation pattern could be regarded as normal and healthy.

Second, the ‘heavy-user’ pattern features a significant decrease in HbO and an increase in HbR in BA 9. However, a remarkable increase in HbR (since 8th second) and HbO (since 12th second) was also found. According to the hemodynamic rule, the concentration of HbO is expected to rise after the activation of the prefrontal cortex due to the higher blood flow. In contrast, HbR is washed out, and its concentration should decrease [23]. Therefore, the ‘heavy-user’ pattern has two unexpected features observed in the DCCS task. The first unusual feature is the significant decrease in HbO before the 12th second, reflecting BA 9 might not be activated during this period. The second unusual feature is the synchronous increase in HbO and HbR after the 12th second, which is inconsistent with the hemodynamic rule [24]. During activation of BA 9, there should be an increase in HbO but a decrease in HbR [24]. However, this unexpected synchronous increase in HbO and HbR was also found by Nguyen et al. during epileptic seizures [25]. They found that an initial decrease in HbR was followed by its increase, indicating an increase in oxygen metabolism is not sufficiently compensated during the epileptic seizures. Therefore, we tend to suggest that this ‘heavy-user’ activation pattern might be abnormal and very likely unhealthy, demonstrating the negative impact of tablet use on young children’s executive function. However, future studies are needed to further explore this synchronous increase phenomenon and its hemodynamic mechanisms.

## 5. Conclusions, Limitations, and Implications

This study adopted fNIRS technology to explore the impact of heavy use of tablets on preschoolers’ executive function. It found that the ‘non-user’ group outperformed the ‘heavy-user’ with a significantly higher correct rate in the DCCS task. The ‘non-user’ brain activation pattern in BA 9 is normal and healthy, whereas the ‘heavy-user’ pattern is not normal and deserves further studies.

However, this study did have four major limitations. First, fNIRS data acquisition could have been disturbed by movement during the DCCS task. Fortunately, the prefrontal cortex is associated with little movement and consists mainly of executive function. In the future, EEG-fNIRS signals should be simultaneously collected to further understand the activation pattern in BA 9 for those heavy users. The second limitation is the small sample size. Due to the unexpected lockdown caused by the COVID-19 outbreak in China, we had to stop the experiment after testing the 38 samples. This problem might have limited the significance of some of the statistical comparisons in this study. Third, although the total sample was balanced in gender, the different groups had an unbalanced allocation of boys and girls. Future studies should consider this variable if aiming to examine the gender difference in brain activation. Fourth, although there is evidence of a brain activation pattern similar to that of seizures, there is no indication of any permanent damage in BA 9 for those heavy users. All these limitations jointly imply that further studies are necessary to study the consequences of heavy use of tablets, using both longitudinal and EEG-NIRS data.

## Figures and Tables

**Table 1:** Comparison of behavioral performance (correct rate) in the DCCS task.

	Non-Users Mean (*SD*)	Heavy Users Mean (*SD*)	*t*	*p*
Age	5.03 (0.41)	4.80 (0.68)	0.850	0.404
DCCS Score	1 (0)	0.922 (0.14)	2.256	0.039 *

*Note:* * *p* < 0.05.

**Table 2:** Comparison of HbO increase between ‘non-user’ (N_1_ = 8) and ‘heavy-user’ (N_2_ = 16) groups.

	Group	*M*	*SD*	*t*	*p*
Channel 1	Non-user	−0.692	2.045	−0.511	0.615
	Heavy-user	−0.248	1.989		
Channel 2	Non-user	−1.414	1.630	−1.468	0.156
	Heavy-user	−0.105	2.230		
Channel 3	Non-user	−1.024	1.454	0.011	0.991
	Heavy-user	−1.034	2.297		
Channel 4	Non-user	−0.269	1.550	−0.178	0.86
	Heavy-user	−0.146	1.628		
Channel 5	Non-user	−0.439	2.180	−0.318	0.753
	Heavy-user	−0.176	1.767		
Channel 6	Non-user	−0.460	0.698	−1.162	0.258
	Heavy-user	−0.045	0.877		
Channel 7	Non-user	−0.660	1.699	−0.803	0.431
	Heavy-user	0.111	2.423		
Channel 8	Non-user	−1.198	2.068	−0.633	0.533
	Heavy-user	−0.603	2.222		
Channel 9	Non-user	−0.356	0.603	−1.534	0.139
	Heavy-user	−0.015	0.466		
Channel 10	Non-user	0.360	1.667	−0.172	0.865
	Heavy-user	0.465	1.256		
Channel 11	Non-user	0.052	1.322	−1.287	0.211
	Heavy-user	0.761	1.247		
Channel 12	Non-user	0.406	2.823	0.502	0.621
	Heavy-user	−0.044	1.606		
Channel 13	Non-user	0.790	3.030	0.457	0.652
	Heavy-user	0.352	1.702		
Channel 14	Non-user	−0.804	0.984	−0.772	0.448
	Heavy-user	−0.142	2.304		
Channel 15	Non-user	0.444	2.123	0.48	0.636
	Heavy-user	0.037	1.882		
Channel 16	Non-user	1.166	2.019	2.285	0.032 *
	Heavy-user	−0.623	1.699		
Channel 17	Non-user	0.391	1.057	0.724	0.477
	Heavy-user	−0.140	1.920		

*Note*: * *p* < 0.05.

**Table 3:** Comparison of HbR increase between ‘non-user’ (N_1_ = 8) and ‘heavy-user’ (N_2_ = 16) groups.

	Group	*M*	*SD*	*t*	*p*
Channel 1	Non-user	−0.305	1.768	0.604	0.552
	Heavy-user	−0.895	2.452		
Channel 2	Non-user	0.583	2.760	0.307	0.762
	Heavy-user	0.282	1.990		
Channel 3	Non-user	0.304	1.521	0.647	0.524
	Heavy-user	−0.139	1.609		
Channel 4	Non-user	−0.083	2.041	−0.041	0.967
	Heavy-user	−0.045	2.186		
Channel 5	Non-user	0.486	2.210	0.014	0.989
	Heavy-user	0.472	2.306		
Channel 6	Non-user	0.277	0.734	1.339	0.194
	Heavy-user	−0.055	0.479		
Channel 7	Non-user	−0.289	1.472	−0.566	0.577
	Heavy-user	0.178	2.076		
Channel 8	Non-user	−0.482	2.949	0.162	0.872
	Heavy-user	−0.702	3.215		
Channel 9	Non-user	−0.212	0.631	−0.637	0.530
	Heavy-user	−0.062	0.497		
Channel 10	Non-user	0.470	1.988	−0.253	0.802
	Heavy-user	0.634	1.186		
Channel 11	Non-user	1.233	1.060	1.441	0.164
	Heavy-user	0.178	1.914		
Channel 12	Non-user	0.203	1.020	0.329	0.745
	Heavy-user	0.027	1.327		
Channel 13	Non-user	0.683	2.835	−0.042	0.966
	Heavy-user	0.724	1.848		
Channel 14	Non-user	1.378	1.535	1.107	0.280
	Heavy-user	0.536	1.851		
Channel 15	Non-user	1.276	1.816	0.519	0.609
	Heavy-user	0.699	2.846		
Channel 16	Non-user	−0.167	2.602	−0.072	0.943
	Heavy-user	−0.109	1.395		
Channel 17	Non-user	0.391	1.784	0.127	0.900
	Heavy-user	0.298	1.651		

**Table 4:** Predicting HbO increase for the ‘non-user’ and ‘heavy-user’ groups in BA 9.

Group	*β*	Δ*R*^2^	*F*	*t*
Non-user	0.944	0.890	803.452 ***	28.345 ***
‘Heavy-user’	−0.332	0.101	12.171 **	−3.489 **

*Note*: ** *p* < 0.01; *** *p* < 0.001.

**Table 5:** Predicting HbR increase for the ‘non-user’ and ‘heavy-user’ groups in BA 9.

Group	*β*	Δ*R*^2^	*F*	*t*
Non-user	0.761	0.575	134.987 ***	11.618 ***
Heavy-user	0.807	0.647	182.730 ***	13.518 ***

*Note*: *** *p* < 0.001.

## Data Availability

All the data for this study will be available upon request.
